# Supplementary material for: Genome-Scale Analysis of Acetobacterium woodii Identifies Translational Regulation of Acetogenesis
Source: mSystems. 2021 Jul 27;6(4):e00696-21. doi: 10.1128/mSystems.00696-21 (PMC8407422; doi:10.1128/mSystems.00696-21)
Supplement: TEXT S1 [file msystems.00696-21-t0001.pdf]

Transcriptomic landscape of *A. woodii* under fructose and H_2+CO_2 conditions

Total RNA was extracted from the cell cultures at the mid-exponential phase and subjected to Illumina short-read sequencing. We obtained 1.2–4.7 million high-quality sequencing reads, which were uniquely mapped to the reference genome sequence of *A. woodii* (CP002987) (1), corresponding to at least 42.8-fold coverage (Table S1). The correlation coefficient value between the biological replicates demonstrated an experimental reproducibility (Pearson's r = 0.98). Principal component analysis revealed a significant difference in gene expression between heterotrophic and autotrophic growth conditions, indicating a global alteration of cellular functions between the two growth conditions (Fig. S1A–B). RNA-Seq data were then normalised by DEseq2 (2) to estimate differentially expressed genes (DEG) between the two growth conditions with adjusted *P*-value ($Padj < 0.01$), revealing that total 1221 genes were determined as DEGs (Table S2). Expression levels of the selected genes including acetogenesis-related genes were also validated independently by qRT-PCR experiments (Pearson's r > 0.94; Fig. S1E).

Among the DEGs, 674 and 547 genes were up-regulated and down-regulated, respectively, with a dynamic transcriptional expression range from 1.5×10^{-3} to 2.8×10^{3}-fold change in response to autotrophic growth conditions (Fig. S2A and Table S2). To comprehend the functions of the DEGs, their functional categories were determined based on the clusters of orthologous groups (COGs). Most of the up-regulated genes belonged to energy production and conversion (C, 15.97%), amino acid transport and metabolism (E, 8.19%), carbohydrate transport and metabolism (G, 8.06%), coenzyme transport and metabolism (H, 5.28%), and translation, ribosomal structure and biogenesis (J, 5.69%), similar to *Clostridium ljungdahlii* (3) and *Eubacterium limosum* (4). In contrast, most of the down-regulated genes belonged to transcription (10.68%) and cell wall/membrane/envelope biogenesis (3.74%). Furthermore, we identified the DEGs associated with particular metabolic pathways using KEGG pathway enrichment analysis. In general, glycolysis, gluconeogenesis, pentose phosphate pathway, fructose and mannose metabolism, and fatty acid biosynthesis were significantly enriched (Bonferroni corrected $P < 0.0017$; Fig. S2B).

Importantly, *A. woodii* activates genes in the WL pathway under autotrophic growth conditions, where most genes such as those encoding the hydrogen-dependent carbon dioxide reductase (HDCR, Awo_c08190–Awo_c08260), the methyl-branch (Awo_c09260–Awo_c09310), and the carbonyl-branch (Awo_c10670–Awo_c10760) were significantly upregulated (fold changes > 2 and *Padj* < 0.01; Fig. S2C and Table S2). In particular, the highest difference between the two growth conditions was observed for the HDCR genes. The selenium-containing FDH (*FdhF2*) was highly upregulated (fold change > 5.2, $Padj < 8.42 \times 10^{-20}$), whereas selenium-free FDH (*FdhF1*) and the small electron-transferring subunit HycB1 (Awo_c08190–Awo_c08200) were not expressed, indicating that *A. woodii* employed FdhF2 for CO_2 reduction in the presence of selenium in the culture medium. Furthermore, the WL pathway is linked to the chemiosmotic energy conservation system comprising the Rnf complex (Awo_c22010–Awo_c22060) and F_1F_O ATP synthase (Awo_c02140–Awo_c02240) (1, 5). In addition, the electron-bifurcating hydrogenase is required to balance the reduction of the redox carriers NADH and ferredoxin, which are both required for the WL pathway. The Rnf complex has a dual function in redox balancing and energy conservation (6). All these genes

were upregulated (fold change > 2, *Padj* < 0.01), implying their important roles during growth on H_2+CO_2 (Fig. S2C and Table S2). Collectively, the transcriptomic results suggest that the genes encoding acetogenesis as well as gluconeogenesis and pentose phosphate pathway were activated for biomass formation and CO_2 reduction during autotrophic growth (3, 4, 7, 8).

Hydrogen-dependent carbon dioxide reductase

A. woodii utilises the WL pathway comprising methyl- and carbonyl-branch for autotrophic growth for the conversion of two molecules of CO_2 to acetate. The WL pathway is linked to a chemiosmotic energy conservation system consisting of Rnf complex (a ferredoxin–NAD+ oxidoreductase) and F_1F_O ATP synthase (1, 5). Initially, the gene expression (Awo_c08190–Awo_c08260) involved in hydrogen-dependent carbon dioxide reductase (HDCR) revealed the highest difference between the two conditions (Fig. S2C and Table S2). The HDCR system is a unique mechanism for initiating the acetogenesis that directly catalyses the reduction of CO_2 to formate with H_2 or reduced ferredoxin in *A. woodii* (9). Under autotrophic conditions in the presence of Se, selenium-containing (FdhF2) FDH, iron–iron hydrogenase (HydA2), formate dehydrogenase accessory protein (FdhD), and hydrogenase Fe-S subunit (HycB2/3) were up-regulated both at the transcriptional (5.2–10.2-fold changes, *Padj* < 8.42×10^{-20}) and translational (3.0–4.5-fold changes, *Padj* < 5.97×10^{-28}) levels; however, expression of selenium-free FDH (FdhF1) and hydrogenase Fe-S subunit (HycB1; Awo_c08190–Awo_c08200) showed low expression and no difference at the transcription level under both conditions (*Padj* > 0.056; Table S2). Notably, the seleno-containing FDHs exhibited higher catalytic activity relative to the non-selenocysteine FDHs (10). One of the most up-regulated operons (transcription level, > 24.3-fold changes, *Padj* < 6.16^{-78}; translation level, 12.0-fold changes, *Padj* < 9.46^{-105}) anaerobic sulphite reductase (Awo_c17660–Awo_c17690) may play a pivotal role in selenium amino acid metabolism for expressing the selenium-charging FDH (Table S2).

Wood–Ljungdahl pathway

The WL pathway genes were transcriptionally up-regulated under autotrophic growth conditions (Fig. S2C). In. *A. woodii*, the methyl-branch (Awo_c09260 – Awo_c09310) includes the formyl-tetrahydrofolate (THF) synthetase (FTHFS), formyl-THF cyclohydrolase (MTHFC), methylene-THF dehydrogenase (MTHFD) and methylene-THF reductase (MTHFR). These methyl-branch enzymes catalyse the reduction of the formyl-group to the methyl-group (1, 5, 11). Under autotrophic growth conditions, expression of most of the genes involved in the methyl-branch was significantly up-regulated at transcriptional (2.0–7.5 fold-changes, *Padj* < 1.18×10^{-7}) and translational (1.5–2.1 fold-changes, *Padj* < 1.96×10^{-6}) levels, except *fsh1* (Awo_c09260; Fig. S2C and Table S2). Similarly, all genes encoding the carbonyl-branch (Awo_c10670–Awo_c10760) were significantly up-regulated at the transcription level (1.4–8.5-fold changes, *Padj* < 2.48×10^{-14}) during autotrophy except *cooc1* (Awo_c10670; Fig. S2C and Table S2). In particular, the ACS/CODH (carbon monoxide dehydrogenase/acetyl-CoA synthase) and MET/CoFeSP gene cluster (Awo_c10710–Awo_c10760) containing *acsA*–*acsD* genes was highly up-

regulated (3.1–8.5-fold changes, *Padj* < 2.48×10^{-14}) among the carbonyl-branch genes; however, surprisingly, none of the genes retained a similar (*Padj* < 0.85) or lower RPF level under autotrophic growth condition (fold changes < 0.78, *Padj* < 0.61×10^{-2}; Table S2).

Energy conservation system

We observed that the expression of the genes encoding energy conservation associated enzymes were transcriptionally up-regulated under autotrophic growth conditions (Fig. S2C and Table S2). In *A. woodii*, the WL pathway is linked with the energy conservation system comprising F_1F_O-ATP synthase and Rnf complex (1, 5). For energy conservation, first, ferredoxin and NAD+ are reduced with two H_2 by the electron-bifurcating hydrogenase (12). *A. woodii* genome contains multimeric [FeFe]-hydrogenase genes (hydCEDBA1) comprising five genes (Awo_c26970–Awo_c27010), which were highly activated at both transcription (2.0–7.7 fold-changes, *Padj* < 2.26×10^{-6}) and translation (2.3–3.7 fold-changes, *Padj* < 2.24×10^{-20}) levels during acetogenesis with H_2+CO_2 (Fig. S2C and Table S2). Collectively, electron-bifurcating hydrogenase and the hydrogenase of the HDCR complex, which catalyse hydrogen activation, were significantly up-regulated during H_2-dependent autotrophic growth. Subsequently, reduced ferredoxin is used for reductive reactions of WL pathway and the establishment of sodium ion gradient via the Rnf complex (Awo_c22010–Awo_c22060). Expression of the Rnf complex was significantly up-regulated at the transcription level (1.9–2.9 fold-changes, *Padj* < 2.26×10^{-6}) under autotrophic growth conditions, except *rnfC1* (Awo_c22060), whereas these genes were retained at a similar (*Padj* < 0.27) or slightly lower stage (0.68–0.74 fold-changes, *Padj* < 8.12×10^{-3}) in RPF level (Fig. S2C and Table S2). The generated sodium ion gradient is used for producing ATP via F_1F_O ATP synthase complexes. *A. woodii* has a particular membrane-bound F_1F_O ATP synthase (*atp*IB$E_1E_2E_3$FHAGDC, Awo_c02140–Awo_c02240), which has more c-subunits than a typical bacterial ATP synthase (*atp*IBEFHAGDC) (1, 13). The gene clusters of F_1F_O ATP synthase were significantly up-regulated at the transcription level (1.8–6.9 fold-changes and *Padj* < 1.72×10^{-4}) under autotrophic growth conditions, whereas the translation levels of these genes were retained at a similar or lower level (0.69–0.89 fold-changes; Table S2). Interestingly, we observed that the transcription of ATP synthase operon commenced from the *atpI* gene body. TSS position of ATP synthase operon was in accordance with our dRNA-seq and rapid amplification of sequences from the 5′-ends of mRNAs (5′RACE) validation (Fig. S5C and Table S2). Although AtpI might be essential for hybrid rotor assembly in *E. coli* (14), the functional role was not exactly determined in *A. woodii*. Thus, most of the energy conservation related genes are highly activated at the transcription level during acetogenesis with H_2+CO_2. The autotrophic-specific Rnf expression was detected in *E. limosum* (4), *C. ljungdahlii* (15) and *C. autoethanogenum* (7); however, the Ribo-Seq analysis presented direct evidence that *A. woodii* regulates the expression of Rnf complex and ATP synthase at the translation level.

Central carbon metabolic pathway

The expression of several central carbon metabolic pathways was also regulated

during autotrophic growth. Initially, the rate-limiting enzymes for gluconeogenesis, that is fructose-1,6-bisphosphatase (Awo_c08060) and pyruvate phosphate dikinase (PPDK) genes, were significantly up-regulated both at transcription (3.0–9.7 fold-changes, $Padj < 4.14 \times 10^{-12}$) and translation (2.6–6.8 fold-changes, $Padj < 3.84 \times 10^{-27}$) levels, suggesting that gluconeogenesis is enhanced under autotrophic growth conditions. In contrast, glycolysis-specific genes, such as 6-phosphofructokinase (PFK, Awo_c12790) and pyruvate kinase (PK) Awo_c12800 are significantly down-regulated at the translation level (0.5–0.6 fold-changes, $Padj < 1.08 \times 10^{-13}$) under autotrophic growth conditions, which is one of the key regulatory and rate-limiting steps of glycolysis. In the case of non-oxidative pentose phosphate pathway, all genes encoding trans-ketolase (TKT) and trans-aldolase (TAL) acting as a major enzyme in non-oxidative pentose phosphate pathway are highly up-regulated, and overall expression pattern of the pentose phosphate pathway is similar for both transcription and translation levels (Fig. S2C and Table S2). Pyruvate:ferredoxin oxidoreductase (PFOR) is also up-regulated at both transcription (5.9–15.1 fold-changes and $Padj < 4.60 \times 10^{-32}$) and translation (2.9–8.4 fold-changes, $Padj < 2.35 \times 10^{-35}$) levels under autotrophic growth conditions. The PFOR is central to cellular carbon synthesis as it catalyses the reductive carboxylation of acetyl-CoA to pyruvate during autotrophic growth by the WL pathway (16). For the branched TCA cycle, we did not observe the up-regulated or considerable change in the transcript abundance between the two growth conditions (Fig. S2C).

References

1. Poehlein A, Schmidt S, Kaster A-K, Goenrich M, Vollmers J, Thürmer A, Bertsch J, Schuchmann K, Voigt B, Hecker M, Daniel R, Thauer RK, Gottschalk G, Müller V. 2012. An ancient pathway combining carbon dioxide fixation with the generation and utilization of a sodium ion gradient for ATP synthesis. PLoS ONE 7:e33439-8.
2. Love MI, Huber W, Anders S. 2014. Moderated estimation of fold change and dispersion for RNA-seq data with DESeq2. Genome Biol 15:550.
3. Tan Y, Liu J, Chen X, Zheng H, Li F. 2013. RNA-seq-based comparative transcriptome analysis of the syngas-utilizing bacterium *Clostridium ljungdahlii* DSM 13528 grown autotrophically and heterotrophically. Mol Biosyst 9:2775-2784.
4. Song Y, Shin J, Jin S, Lee J-K, Kim DR, Kim SC, Cho S, Cho B-K. 2018. Genome-scale analysis of syngas fermenting acetogenic bacteria reveals the translational regulation for its autotrophic growth. BMC Genomics 19:837.
5. Schuchmann K, Müller V. 2014. Autotrophy at the thermodynamic limit of life: a model for energy conservation in acetogenic bacteria. Nat Rev Microbiol 12:809-821.
6. Westphal L, Wiechmann A, Baker J, Minton NP, Müller V. 2018. The Rnf complex is an energy-coupled transhydrogenase essential to reversibly link cellular NADH and ferredoxin pools in the acetogen *Acetobacterium woodii.* J Bacteriol 200:602.
7. Marcellin E, Behrendorff JB, Nagaraju S. 2016. Low carbon fuels and commodity chemicals from waste gases–systematic approach to understand energy metabolism in a model acetogen. Green Chem 18:3020-3028.
8. Aklujkar M, Leang C, Shrestha PM, Shrestha M, Lovley DR. 2017.

Transcriptomic profiles of *Clostridium ljungdahlii* during lithotrophic growth with syngas or H2 and CO2 compared to organotrophic growth with fructose. Sci Rep 7:232.

9. Schuchmann K, Muller V. 2013. Direct and reversible hydrogenation of CO2 to formate by a bacterial carbon dioxide reductase. Science 342:1382-1385.
10. Stadtman TC. 1991. Biosynthesisand Function of Selenocysteine-containing Enzymes. J Biol Chem 266:16257-16260.
11. Bertsch J, Öppinger C, Hess V, Langer JD, Müller V. 2015. Heterotrimeric NADH-oxidizing methylenetetrahydrofolate reductase from the acetogenic bacterium *Acetobacterium woodii*. J Bacteriol 197:1681-1689.
12. Schuchmann K, Müller V. 2012. A bacterial electron-bifurcating hydrogenase. J Biol Chem 287:31165-31171.
13. Matthies D, Zhou W, Klyszejko AL, Anselmi C, Yildiz O, Brandt K, Müller V, Faraldo-Gómez JD, Meier T. 2014. High-resolution structure and mechanism of an F/V-hybrid rotor ring in a Na^+-coupled ATP synthase. Nat Commun 5:5286.
14. Brandt K, Müller DB, Hoffmann J, Hübert C, Brutschy B, Deckers-Hebestreit G, Müller V. 2013. Functional production of the Na+ F1F(O) ATP synthase from Acetobacterium woodii in Escherichia coli requires the native AtpI. J Bioenerg Biomembr 45:15-23.
15. Richter H, Molitor B, Wei H, Chen W, Aristilde L, Angenent LT. 2016. Ethanol production in syngas-fermenting *Clostridium ljungdahlii* is controlled by thermodynamics rather than by enzyme expression. Energy Environ Sci 9:2392-2399.
16. Furdui C, Ragsdale SW. 2000. The role of pyruvate ferredoxin oxidoreductase in pyruvate synthesis during autotrophic growth by the Wood-Ljungdahl pathway. J Biol Chem 275:28494-28499.
