# Supplementary figures and images for: Genome-Scale Analysis of Acetobacterium woodii Identifies Translational Regulation of Acetogenesis
Source: mSystems. 2021 Jul 27;6(4):e00696-21. doi: 10.1128/mSystems.00696-21 (PMC8407422; doi:10.1128/mSystems.00696-21)

**A**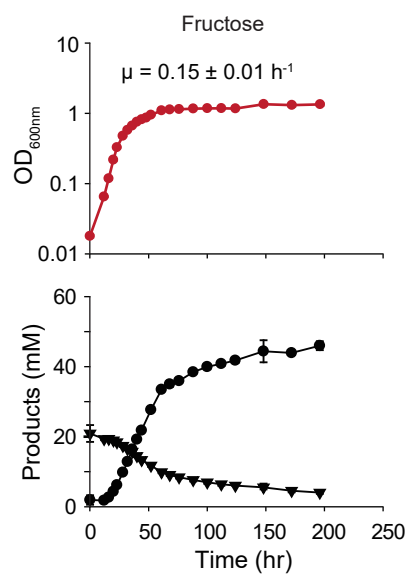**B**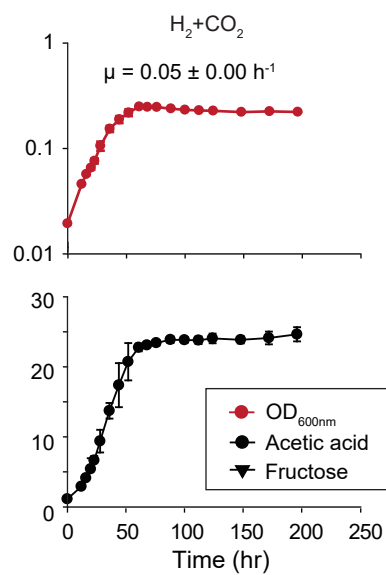**C**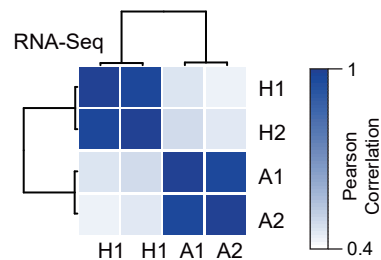**D**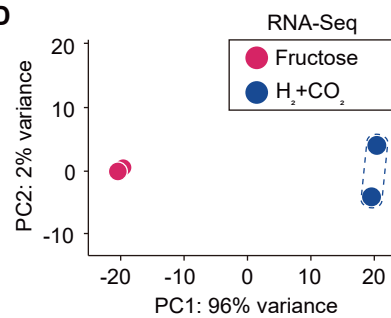**E**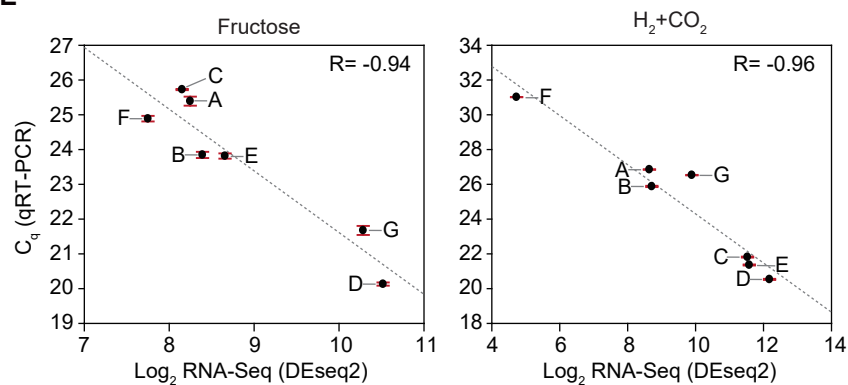**F**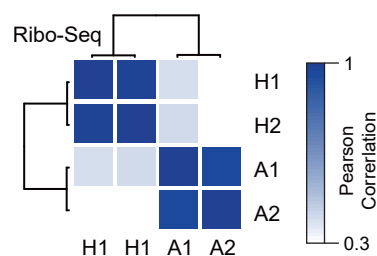**G**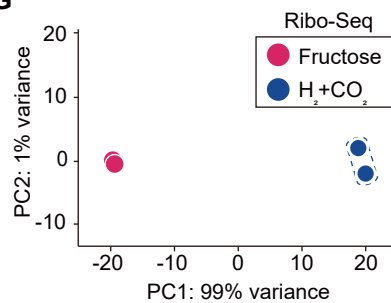**H**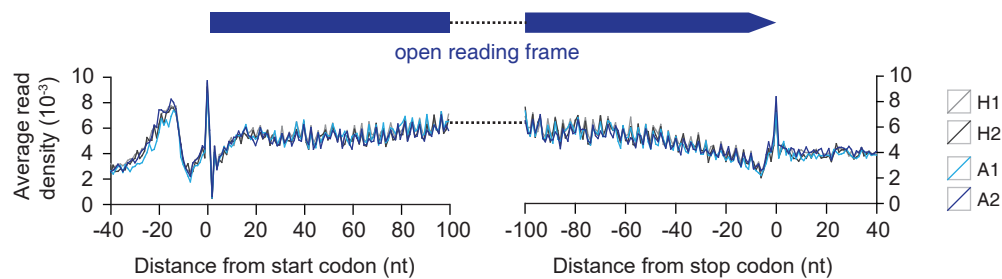

Supplement: FIG S1 [file msystems.00696-21-sf001.pdf]

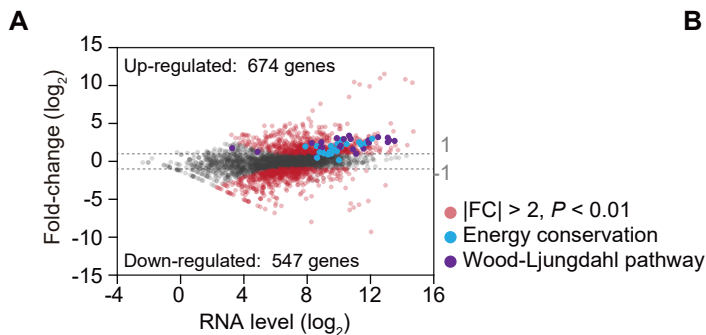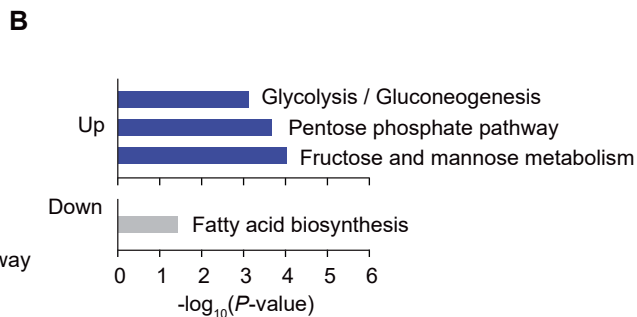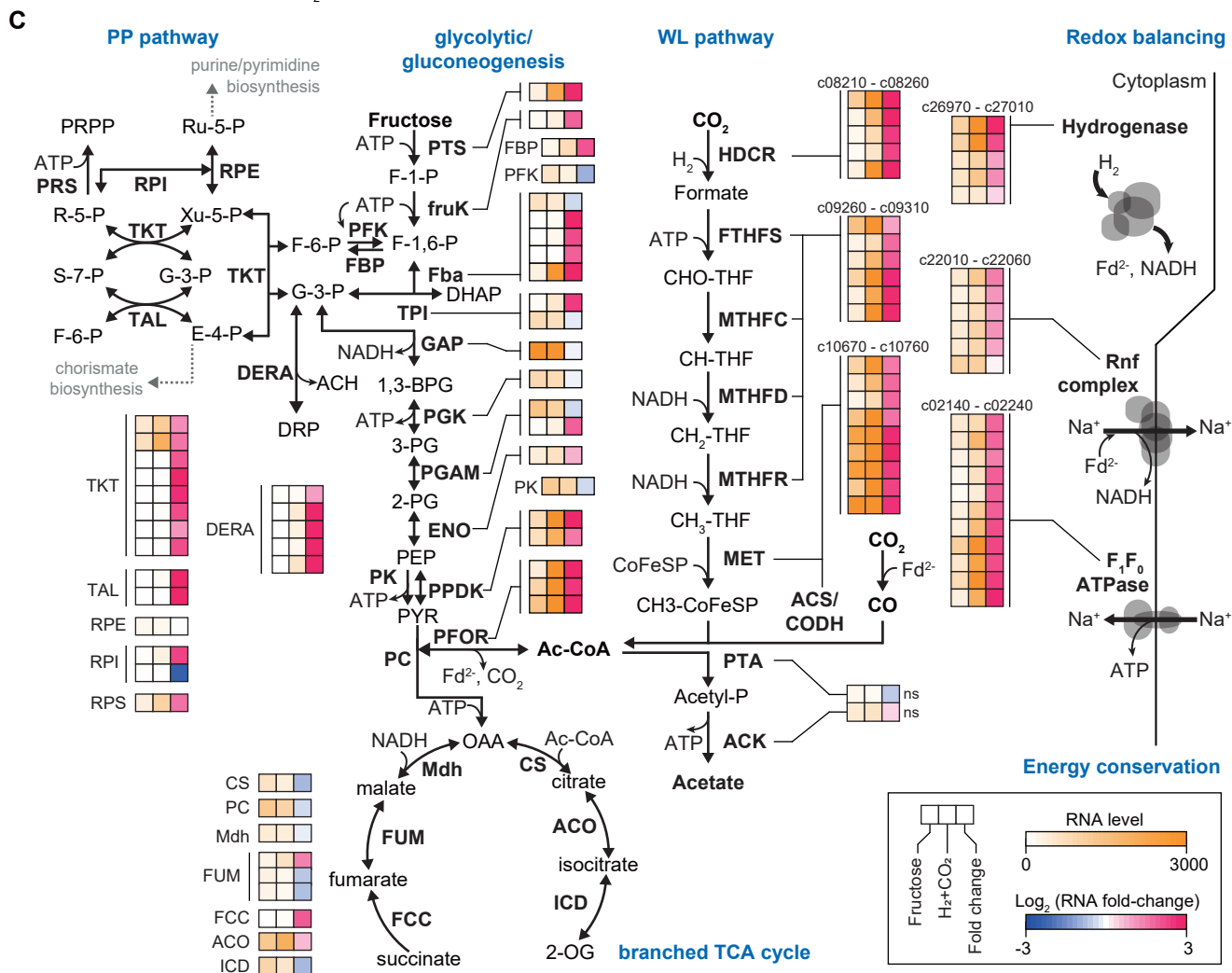

Supplement: FIG S2 [file msystems.00696-21-sf002.pdf]

**A**

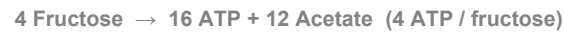

## B

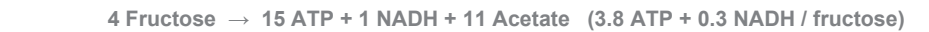

**C**

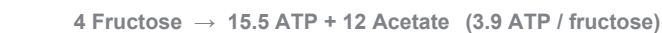

Supplement: FIG S4 [file msystems.00696-21-sf004.pdf]

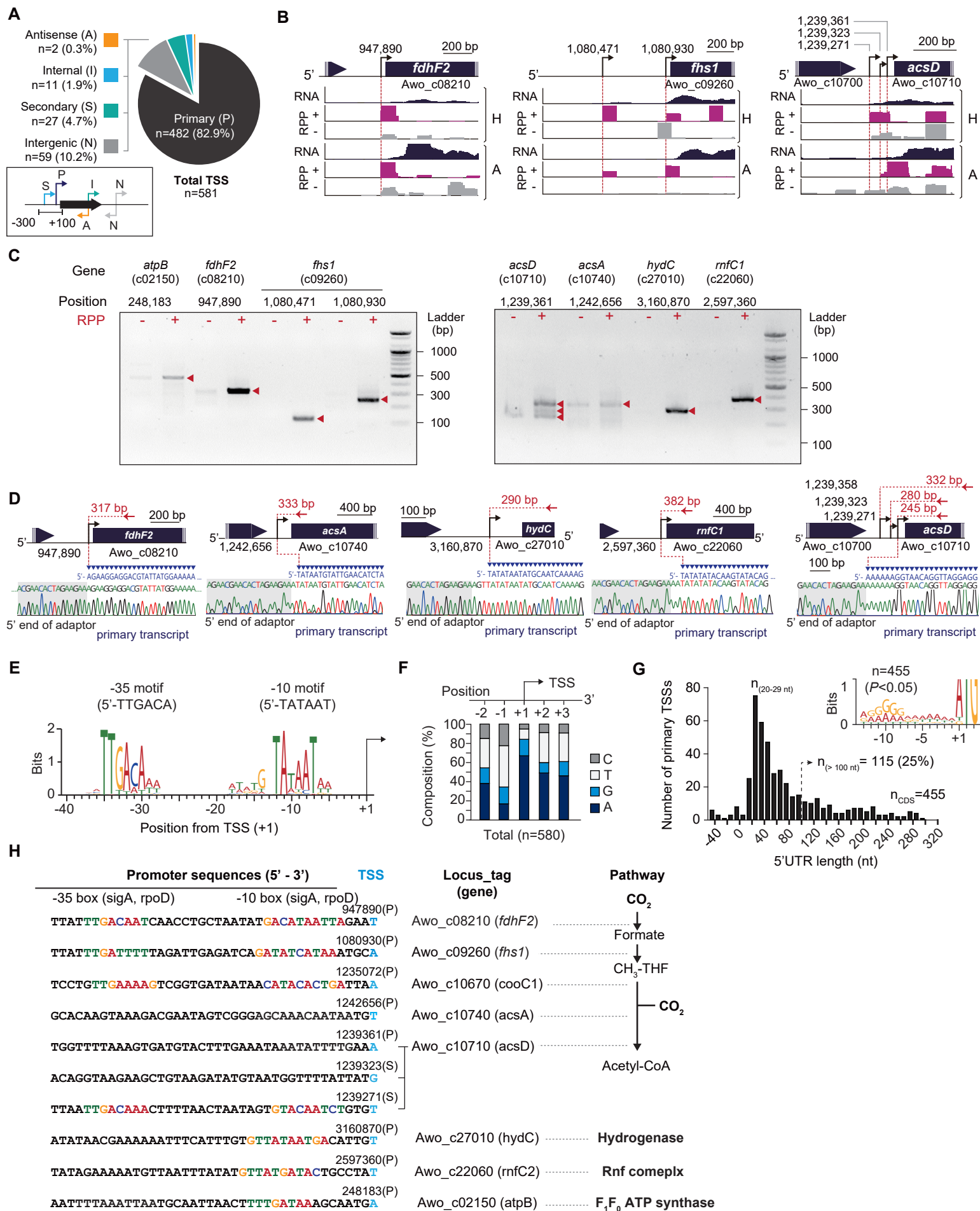

Supplement: FIG S5 [file msystems.00696-21-sf005.pdf]
