# Supplementary material for: Genome-Scale Analysis of Acetobacterium woodii Identifies Translational Regulation of Acetogenesis
Source: mSystems. 2021 Jul 27;6(4):e00696-21. doi: 10.1128/mSystems.00696-21 (PMC8407422; doi:10.1128/mSystems.00696-21)
Supplement: TABLE S1 [file msystems.00696-21-st001.pdf]

Library	Sample	Raw data		Quality and adapter trimming		Genome mapping (unique)			
		Reads	Len.	Remaining reads	Len.	Mapped Reads	# of bases	Mapped (%)	Cov.
**RNA-Seq**	Heterotroph1	1,411,725	150	1,348,949	142.2	1,216,393	173,191,739	86.2	42.8
	Heterotroph2	1,788,006	150	1,696,513	142.2	1,534,261	218,539,243	85.8	54.0
	Autotroph1	5,800,707	150	5,655,918	149.84	4,708,159	705,704,589	81.2	174.5
	Autotroph2	4,443,182	150	4,326,000	149.05	3,750,712	559,149,623	84.4	138.2
**dRNA-Seq**	H1_RPP+	9,790,993	101	5,332,815	98.2	4,197,209	420,657,989	42.9	104.0
	H1_RPP-	10,719,079	101	9,686,711	99.9	8,897,371	892,437,786	83.0	220.6
	H2_RPP+	10,220,613	101	7,309,099	98.4	5,102,158	510,436,818	49.9	126.2
	H2_RPP-	10,130,114	101	8,103,699	98.3	6,588,945	659,747,224	65.0	163.1
	A1_RPP+	9,096,793	101	8,158,305	98.8	7,320,536	733,184,147	80.5	181.3
	A1_RPP-	11,297,012	101	8,711,449	91.6	6,022,826	601,518,319	53.3	148.7
	A2_RPP+	10,387,256	101	7,340,683	97.5	6,799,227	680,828,312	65.5	168.3
	A2_RPP-	10,957,336	101	6,549,255	91.4	4,870,750	484,775,506	44.5	119.9
**Library**	**Sample**	**Raw data**		**Quality and adapter trimming**		**Genome mapping (tRNA and rRNA region masked genome)**			
		**Reads**	**Len.**	**Remaining reads**	**Len.**	**Mapped Reads**	**# of bases**	**Mapped (%)**	**Cov.**
**Ribo-Seq**	Heterotroph1	249,172,796	51	221,342,687	31.3	34,908,914	1,254,089,172	14.0	310.1
	Heterotroph2	69,476,513	51	61,223,608	29.9	10,251,591	355,253,179	14.8	87.8
	Autotroph1	131,703,279	51	121,595,278	31.7	13,348,277	473,644,767	10.1	117.1
	Autotroph2	139,370,873	51	128,404,895	32.2	18,697,875	689,082,546	13.4	170.4
