# Supplementary material for: Genome-Scale Analysis of Acetobacterium woodii Identifies Translational Regulation of Acetogenesis
Source: mSystems. 2021 Jul 27;6(4):e00696-21. doi: 10.1128/mSystems.00696-21 (PMC8407422; doi:10.1128/mSystems.00696-21)
Supplement: TABLE S3 [file msystems.00696-21-st003.pdf]

| Methods | Oligo name | Length (nt) | Sequence | Target gene | Note |
|---|---|---|---|---|---|
| **dRNA-seq** | 5' RNA adaptor | 33 | ACACUCUUUCCCUACACGACGCUCUUCCGAUCU | N/A | N/A |
| | Solexa_Mul_RT_random primer_N9 (3' random primer) | 43 | GTGACTGGAGTTCAGACGTGTGCTCTTCCGATCT N9 | N/A | N/A |
| | Solexa_Mul_PCR_1 (5' PCR universal primer ) | 58 | AATGATACGGCGACCACCGAGATCTACACTCTTTCC CTACACGACGCTCTTCCGATCT | N/A | N/A |
| | Solexa_Mul_In_XX_long (Index primer) | 64-66 | CAAGCAGAAGACGGCATACGAGATXXXXXXGTGAC TGGAGTTCAGACGTGTGCTCTTCCGATCT | N/A | N/A |
| **qRT-PCR** | WO_qPCR_secA_F | 20 | GGAGCTTGACAGCCGTTTTG | Awo_c28060 | Protein translocase subunit SecA |
| | WO_qPCR_secA_R | 20 | CAAGGGTTTCTCCTTGCGCT | | |
| | WO_qPCR_gyrA_F | 22 | TGAGTGTCATTATAGGTCGGGC | Awo_c00060 | DNA gyrase subunit A GyrA |
| | WO_qPCR_gyrA_B | 20 | ATCCCAATTGGCTCATGGCA | | |
| | WO_qPCR_HydA_F | 20 | AATGTGCCGAAGTGTGTCCA | Awo_c08260 | Iron hydrogenase HydA2 |
| | WO_qPCR_HydA_R | 20 | ATTTCTGTACGCATTGGCCG | | |
| | WO_qPCR_acsD_F | 20 | TTGGGCAGAGTGCTTTGGAA | Awo_c10710 | Corrinoid/iron-sulfur protein, small subunit AcsD |
| | WO_qPCR_acsD_R | 21 | ACAGCAAGAAATCAGCACCTG | | |
| | WO_qPCR_metF_F | 20 | TGCGTACTGGTTCATTGGCA | Awo_c09310 | Methylenetetrahydrofolate reductase large subunit MetF |
| | WO_qPCR_metF_R | 21 | GGCAATACGATTTCGATCCCG | | |
| | WO_qPCR_pc_F | 20 | GGATAAACGGTCCGGTGGAA | Awo_c26160 | Pyruvate carboxylase PC |
| | WO_qPCR_pc_R | 20 | CCATAACCGGGATGGATTGC | | |
| | WO_qPCR_lctB_F | 20 | AGAACAATTAGGCGGGACCA | Awo_c08710 | Electron transfer flavoprotein beta-subunit EtfB |
| | WO_qPCR_lctB_R | 20 | CCTCATCAGCGCCCATGTAA | | |
| | EL_qPCR_ptsF_F | 20 | GCCAGAGAGGAACAGGGAAC | ELIM_c2548 | Fructose specific PTS system ptsF in *E. limosum* |
| | EL_qPCR_ptsF_R | 20 | TCCTTGCTCACCATGACAGC | | |
| | EL_qPCR_gyr_F | 20 | TCGGACAGGGTAACTTTGGC | ELIM_c0629 | DNA gyrase gyr in *E. limosum* |

| | | | | | |
|---|---|---|---|---|---|
| | EL_qPCR_gyr_R | 20 | CAGCATTTCCAGCGCGATTT | | |
| | qPCR_gcvH3_F | 20 | GCCCAACATTCACTGGGAGA | Awo_c32790 | Heterologous expression of gcvH in *E. limosum* |
| | qPCR_gcvH3_R | 20 | TCCGAAGCCGCTTTTACTGA | | |
| | qPCR_gcvT_F | 20 | GGTTACACCGGGGAACCATT | Awo_c32790 | Heterologous expression of gcvH in *E. limosum* |
| | qPCR_gcvT_R | 20 | CCATCGCTCCCAGTTCCAAT | | |
| | qPCR_gcvPA_F | 20 | CGGGGAAAAATGCCAACCTG | Awo_c32790 | Heterologous expression of gcvH in *E. limosum* |
| | qPCR_gcvPA_R | 20 | ATCAGGTTTCCAACCGCACT | | |
| | qPCR_gcvPB_F | 20 | GCCGCAGTGGTAGGATTTGA | Awo_c32780 | Heterologous expression of gcvH in *E. limosum* |
| | qPCR_gcvPB_R | 20 | GGCGATCTCATCGCTCATCA | | |
| **5'RACE** | FW.5bt (Short RNA adapter) | 15 | ACGGACUAGAAGAAA | N/A | N/A |
| | FR.RNA5 (Second RNA adapter) | 38 | AUAUGCGCGAAUUCCUGUAGAACGAACACUAGAAGAAA | N/A | N/A |
| | FR.DNA5 | 20 | GCGCGAATTCCTGTAGAACG | N/A | N/A |
| | 5RACE_2105_R | 20 | AATGACTTGGGCCTTCCCAG | Awo_c02150 | F-type ATP synthase subunit A |
| | 5RACE_2105_R | 21 | GCTGATCGCTTCATCCCACT | Awo_c08210 | Formate dehydrogenase FdhF2 |
| | nc0930_R | 21 | TCTTGTTCCCGTTACCCAGC | Awo_c09260 | Non-coding region |
| | 9260_R | 20 | TCAGGATCAGTCGCGCTTTT | | Formyl-THF synthetase Fhs1 |
| | 10710_R | 20 | CTCTGCCCAATCTTCTGGGG | Awo_c10710 | Corrinoid/iron-sulfur protein, small subunit AcsD |
| | 10740_R | 20 | GGCAACATACCCCTGCTTCT | Awo_c10740 | Carbon monoxide dehydrogenase AcsA |
| | 27010_R | 20 | ACGTTGCAACACCGTAGACT | Awo_c27010 | Iron hydrogenase HydC |
| | 22060_R | 20 | CTCGATCACCCGATGCATGT | Awo_c22060 | Electron transport complex protein RnfC1 |
| **Antibody production** | ELIM_c0957_F | 40 | AGGAGATATACCATGGGTTTCAAATCAGACATCGAAATTG | ELIM_c0957 | Formate--tetrahydrofolate ligase Fhs, FTHFS |
| | ELIM_c0957_R | 37 | GGTGGTGGTGCTCGAGGAATAAGCCGGAGATTACGCC | | |
| | ELIM_c0959_F | 35 | AGGAGATATACCATGGCAGCAAAATTATTAAGTGG | ELIM_c0959 | Bifunctional protein FolD, MTHFD |

| | | | | | |
|---|---|---|---|---|---|
| | ELIM_c0959_R | 36 | GGTGGTGGTGCTCGAGTAAATTATTCTGCTGTTTGC | | |
| | ELIM_c1650_F | 40 | AGGAGATATACCATGCCATTCAAAAAAGCAGAACAA AAGT | ELIM_c1650 | acetyl-CoA decarbonylase/synthase complex subunit delta AcsD |
| | ELIM_c1650_R | 41 | GGTGGTGGTGCTCGAGACCTACTAACTCTTTGATAA AGTTT | | |
| | ELIM_c1652_F | 40 | AGGAGATATACCATGGCAAAATTTATGGTTATCGGT GAAA | ELIM_c1652 | 5-methyltetrahydrofolate corrinoid/iron sulfur protein methyltransferase AcsE |
| | ELIM_c1652_R | 38 | GGTGGTGGTGCTCGAGGAATTCCAGAACGGAGTCT GCA | | |
| | ELIM_c1655_F | 40 | AGGAGATATACCATGGAACGTAAGACTTATAATCTT TTTG | ELIM_c1655 | acetyl-CoA synthase AcsB |
| | ELIM_c1655_R | 39 | GGTGGTGGTGCTCGAGCATAATTGGATCCATTTCCA GTG | | |
| **Plasmid construction** | tetO1_F | 68 | CCGGGGATCCTCTATTGAAACTCTATCATTGATAGA GTATAATATCTTTGTTCATTAGAGCGATAAAC | N/A | ptetO1 (107 bp) promoter fragment |
| | tetO1_R | 62 | AGGGCGATCGGGCTCCATGGTATTCCCTCTCAAATT CAAGTTTATCGCTCTAATGAACAAAG | | |
| | ptet_tetR_F | 40 | ATGATTACGAATTCGTTCTCTATCACTGATAGGGAG TGGT | N/A | TetR gene (728 bp) from pdCas9 plasmid (Addgene plasmid # 46569) |
| | ptet_tetR_R | 40 | TTTCAATAGAGGATCTTAAGACCCACTTTCACATTTA AGT | | |
| | GCS_IF_F | 40 | AGAGGGAATACCATGATGAAAATTATTGAAGGTCTG AAAT | N/A | A DNA fragment (6117 bp) containing gcvHTP genes for construction of pJIR-GCS |
| | GCS_IF_R | 37 | CTGTTGGGAAGGGCGCGCGGATCTTATTTTGATCAT T | | |
| | GCS_IF_NoP_F | 36 | AAGAGGCCCGCACCGGCGCGGATCTTATTTTGATC A | N/A | A DNA fragment (6131 bp) containing gcvHTP genes for construction of pJIR-NoP |
| | GCS_IF_NoP_R | 37 | CTGTTGGGAAGGGCGAGAGGGAATACCCATGAAAA TT | | |
