# Supplementary material for: Genome-Scale Analysis of Acetobacterium woodii Identifies Translational Regulation of Acetogenesis
Source: mSystems. 2021 Jul 27;6(4):e00696-21. doi: 10.1128/mSystems.00696-21 (PMC8407422; doi:10.1128/mSystems.00696-21)
Supplement: FIG S3 [file msystems.00696-21-sf003.pdf]

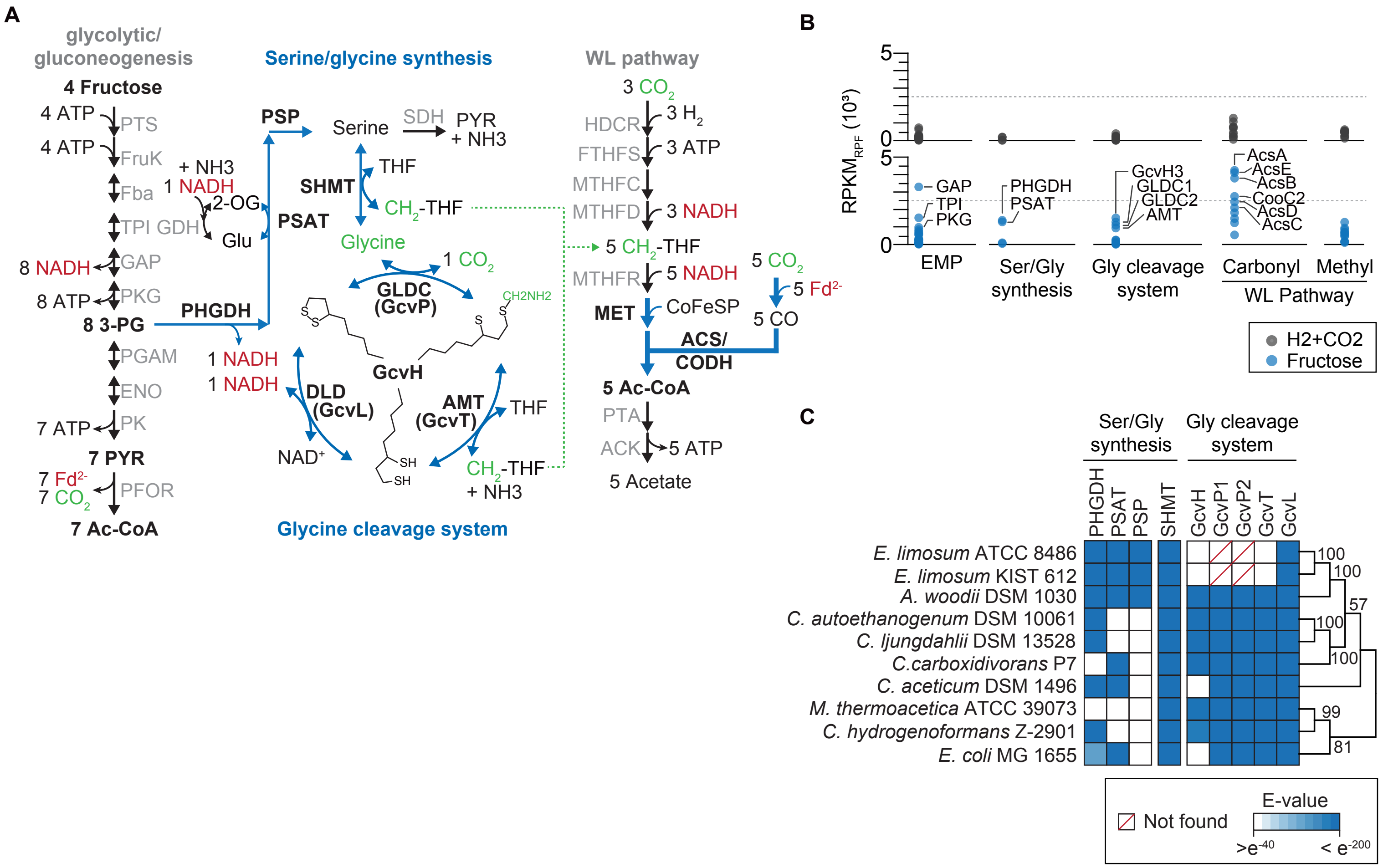
A
glycolytic/ gluconeogenesis
Serine/glycine synthesis
WL pathway
4 Fructose
4 ATP
PTS
FruK
Fba
TPI GDH
GAP
PKG
8 NADH
8 ATP
8 3-PG
PGAM
ENO
PK
7 ATP
7 PYR
7 Fd2-
7 CO2
PFOR
7 Ac-CoA
+ NH3
1 NADH
2-OG
Glu
PSP
PSAT
PHGDH
1 NADH
1 NADH
Serine
SDH
PYR + NH3
SHMT
THF
CH2-THF
Glycine
1 CO2
GLDC (GcvP)
CH2NH2
GcvH
DLD (GcvL)
NAD+
AMT (GcvT)
THF
CH2-THF + NH3
SH
SH
Glycine cleavage system
3 CO2
HDCR
3 H2
FTHFS
3 ATP
MTHFC
MTHFD
3 NADH
5 CH2-THF
MTHFR
5 NADH
5 CO2
5 Fd2-
MET
CoFeSP
5 CO
ACS/ CODH
5 Ac-CoA
PTA
ACK
5 ATP
5 Acetate
B
RPKMRPF (10³)
5
0
GAP
TPI
PKG
PHGDH
PSAT
GcvH3
GLDC1
GLDC2
AMT
AcsA
AcsE
AcsB
CooC2
AcsD
AcsC
EMP
Ser/Gly synthesis
Gly cleavage system
Carbonyl
Methyl
WL Pathway
H2+CO2
Fructose
C
Ser/Gly synthesis
Gly cleavage system
PHGDH
PSAT
PSP
SHMT
GcvH
GcvP1
GcvP2
GcvT
GcvL
E. limosum ATCC 8486
E. limosum KIST 612
A. woodii DSM 1030
C. autoethanogenum DSM 10061
C. ljungdahlii DSM 13528
C.carboxidivorans P7
C. aceticum DSM 1496
M. thermoacetica ATCC 39073
C. hydrogenoformans Z-2901
E. coli MG 1655
100
100
57
100
100
99
81
Not found
E-value
>e-40
< e-200
